# Phantom imaging demonstration of positronium lifetime with a long axial field-of-view PET/CT and ^124^I

**DOI:** 10.1186/s40658-025-00790-z

**Published:** 2025-08-26

**Authors:** Lorenzo Mercolli, William M. Steinberger, Narendra Rathod, Maurizio Conti, Paweł Moskal, Axel Rominger, Robert Seifert, Kuangyu Shi, Ewa Ł. Stępień, Hasan Sari

**Affiliations:** 1 Department of Nuclear Medicine, Inselspital, Bern University Hostpital, University of Bern, Bern, Switzerland; 2 ARTORG Center for Biomedical Engineering Research, University of Bern, Bern, Switzerland; 3 Albert Einstein Center for Fundamental Physics (AEC), Laboratory for High Energy Physics (LHEP), University of Bern, Bern, Switzerland; 4 Siemens Medical Solutions USA, Inc., Knoxville, TN USA; 5 Faculty of Physics, Astronomy and Applied Computer Science, Jagiellonian University, Krakow, Poland; 6 Centre for Theranostics, Jagiellonian University, Krakow, Poland; 7 Siemens Healthineers International AG, Zürich, Switzerland

**Keywords:** Positronium lifetime imaging, Long axial field-of-view PET/CT, ^124^I

## Abstract

****Purpose**:**

Measuring the ortho-positronium (oPs) lifetime in human tissue bears the potential of adding clinically relevant information about the tissue microenvironment to conventional positron emission tomography (PET). Through phantom measurements, we investigate the voxel-wise measurement of oPs lifetime using a commercial long-axial field-of-view (LAFOV) PET scanner.

****Methods**:**

We prepared four samples with mixtures of Amberlite XAD4, a porous polymeric adsorbent, and water and added between 1.12 and 1.44 MBq of ^124^I. The samples were scanned in two different setups: once with a couple of centimeters between each sample (15 min scan time) and once with all samples taped together (40 min scan time). For each scan, we determine the oPs lifetime for the full samples and at the voxel level. The voxel sizes under consideration are 10.0^3^ mm^3^, 7.1^3^ mm^3^ and 4.0^3^ mm^3^.

****Results**:**

Amberlite XAD4 allows the preparation of samples with distinct oPs lifetime. Using a Bayesian fitting procedure, the oPs lifetimes in the whole samples are 2.52 ± 0.03 ns, 2.37 ± 0.03 ns, 2.27 ± 0.04 ns and 1.82 ± 0.02 ns, respectively. The voxel-wise oPs lifetime fits showed that even with 4.0^3^ mm^3^ voxels the samples are clearly distinguishable and a central voxels have good count statistics. However, the situation with the samples close together remains challenging with respect to the spatial distinction of regions with different oPs lifetimes.

****Conclusions**:**

Our study shows that positronium lifetime imaging on a commercial LAFOV PET/CT is feasible using ^124^I.

## Introduction

Ortho-positronium (oPs), the spin 1 state of an electron-positron bound state, has a significantly longer lifetime in vacuum than the spin 0 state, which is called para-positronium (pPs). The lifetime of pPs is too short in order to interact significantly with the environment [1]. However, oPs has a lifetime of about $142\, \text{ ns }$ in vacuum and it can therefore undergo different interactions with surrounding atoms and molecules (see e.g. Refs. [1–4]). In particular, the oPs’ positron can annihilate with an environmental electron, and thereby, the oPs lifetime can be significantly shortened. This so-called pick-off process makes the oPs lifetime dependent on the atomic and molecular structure of the surrounding material. oPs lifetime is also shortened by a spin exchange process depending on the concentration of oxygen molecules [1, 5, 6]. In vacuum oPs decays into three photons, while in matter due to the pick-off and conversion processes, it may annihilate also into two photons. In principle, both decays can be used for measuring the lifetime of oPs properties. However, it was shown that oPs lifetime imaging based on the two-photon annihilation is 300 times more efficient than oPs imaging based on annihilation into three photons [7, 8].

There is a significant interest in the medical domain for oPs lifetime measurements (see e.g. Refs. [1, 3, 4, 7, 9, 10]), mainly driven by the possibility to infer oxygenation levels in human tissue, [5, 6, 11–14], to assess tissue pathology in vivo  [15–19] and to sense pH level and electrolytes within the tissue [13, 20–23]. Recently, the first in vivo positronium images [18] and the first in-vivo measurements of oPs lifetime with clinical positron emission tomography (PET) system [18, 24] were demonstrated. The oPs lifetime has the potential to add diagnostic information, which is currently unavailable or requires additional interventions, such as e.g. biopsy or additional use of hypoxia tracers.

In Refs. [24, 25], we showed that it is possible to do oPs lifetime measurements with a commercial long axial field-of-view (LAFOV) PET scanner [26, 27]. However, in Ref. [24] we also showed that the collection of sufficient count statistics is a major challenge. The voxel-wise determination of oPs lifetime, what is usually called oPs lifetime imaging, has been shown to be feasible only with long-lived radionuclides, long scan times, large voxel sizes or simplifying the fit models [15, 18, 28–31]. Usually, a combination of multiple of these methods is required.

In this report, we demonstrate the feasibility of performing oPs lifetime imaging achieved using a commercial PET/CT scanner under conditions conditions that are broadly representative of those typically encountered in clinical practice with respect to the isotope, activity concentration, scan time and voxel size. As highlighted in Refs. [24, 25, 32], ^124^I possesses favorable characteristics for oPs lifetime imaging [32], it is also well suited for oPs imaging with the Biograph Vision Quadra (Siemens Healthineers, USA) [24, 25] and is routinely used in some departments given the superior imaging characteristics compared to conventional ^131^I imaging. In contrast to other thyroid-directed PET tracers, like [^18^F]tetrafluoroborate, ^124^I PET has usually higher uptake and also enables delayed imaging which can be used for dosimetry applications [33–35]. The extracted oPs lifetimes may serve as a quantitative surrogate for local oxygen tension—a key driver of radio- and chemo-resistance—in metastatic thyroid cancer patients. Using phantom measurements, we identify the conditions under which oPs lifetime imaging is viable and bring to the fore the remaining challenges.

## Materials and methods

**Fig. 1:**
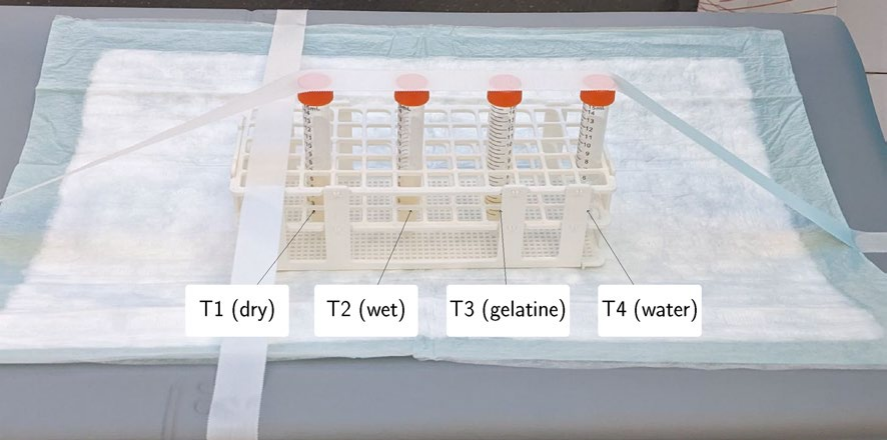
Picture of the experimental setup with the four samples separated from each other

In order to assess the capabilities of Quadra with respect to oPs lifetime imaging, we filled four chemistry tubes with different mixtures of Amberlite XAD4 (Sigma-Aldrich, Co., St. Louis MO, USA) and demineralized water. As shown in Ref. [36], XAD4 allows to vary the oPs lifetime with a simple experimental setup. A relatively low activity of [^124^I]NaI was added to each tube. Table 1 summarizes the details of the sample preparation and Fig. 1 shows the experimental setup. The first tube contained XAD4 that was air-dried for 24 h. T2 contained the wet XAD4 (as it is delivered), while for T2 we added $1 \, \text{ ml }$ of gelatine to $3.5 \, \text{ ml }$ of wet XAD4. In Table 1$m_\text {XAD4}$ is the weight of the wet XAD4 and the gelatine together. To all tubes, about $0.4\, \text{ ml }$ [^124^I]NaI solution was added.

In vitro studies [15, 17] showed that the oPs lifetime in tissues with different oxygenation levels varies up to $\sim 1\, \text{ ns }$. The water sample T4 serves as a reference for the oPs lifetime as this has been measured accurately before (see e.g. Ref. [37]). Hypoxic tissue has a longer oPs lifetime, i.e. tube T1 with the dry XAD4 mimics the hypoxic environments compared to water. However, for clinically relevant studies, measurements should be able to resolve oPs lifetime differences of $\sim 0.15 \, \textrm{ns}$. Therefore, the tubes T2 and T3 have an increasing quantity of liquid in order to simulate decreasing hypoxia in tissue.

Lesions in thyroid cancer patients have shown to have high uptakes of ^124^I in the order of $\textrm{SUVmax}\gtrsim 1000$ and activity concentrations of up to $\sim 70 \, \text{ kBq }/\text{ml }$ (see e.g. Ref. [38]). The tubes in Table 1 have a higher activity concentration of $\sim 250 \text{ kBq }/\text{ml }$. However, much lower activity concentrations would have been difficult to measure in a dose calibrator. In view of patient scans, it would be conceivable to compensate the activity concentration with a longer scan time. The volume and distance of the tubes was chosen such that it could mimic lesions of metastatic differentiated thyroid carcinoma [38].

**Table 1:** Summary of the sample preparation. $0.4\, \text{ ml }$ [^124^I]NaI was added to all tubes. $\rho _\text {tot}$ contains the [^124^I]NaI

Sample	XAD4	$A \; [\text{ MBq}]$	$V_\text {XAD4} \, [\text{ ml}]$	$m_\text {XAD4} \, [\text{ g}]$	$\rho _{\textbf {tot}} \, [\text{ g }/\text{cm}^3]$
T1	Dry	1.12	5.0	3.25	0.67
T2	Wet	1.44	5.5	3.56	0.67
T3	Gelatine	1.14	4.5	3.49	0.79
T4	Deminalized water	1.26	5.0	5.0	1.0

**Fig. 2:**
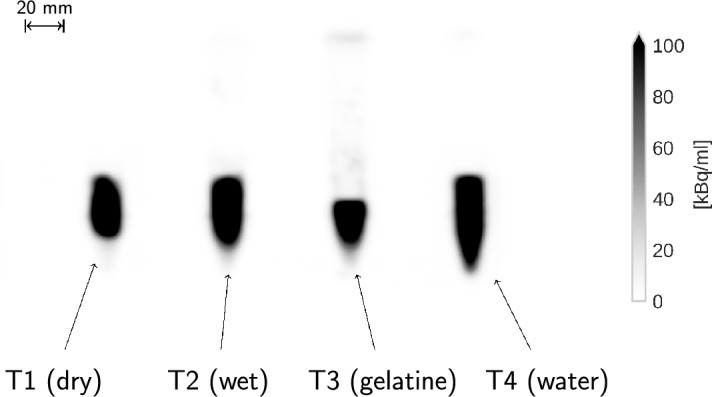
MIP of the coincidence PET images of the four separate tubes

**Fig. 3:**
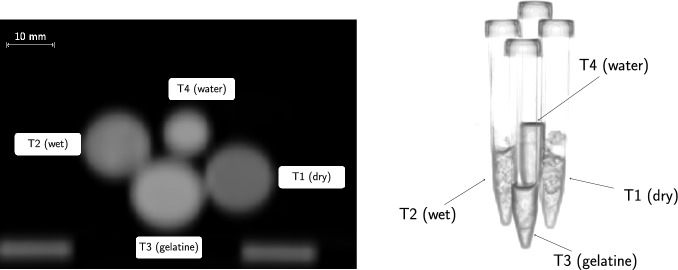
Top view of a CT slice (right) and 3D rendering of the CT (left) of the setup with the four tubes taped together

The samples were measured once with a large distance between them, as shown in Fig. 1, and once taped together (see Fig. 3). In Fig. 2 we show the maximum intensity projection (MIP) of the coincidence PET image of the setup with separated tubes. The experimental setup with the tubes taped together is depicted Fig. 3 with the top view of a CT slice and a 3D rendering from the CT. In the CT images, the voxel size is $1.52\times 1.52\times 1.65 \, \text{ mm}^3$.

The samples were scanned 15 min (separated) and 40 min (taped together) in singles mode. As described in Ref. [25], singles mode in Quadra records all single-crystal interactions into a list mode file. A prototype software was used to sort all three-photon events ($3\gamma \text{ E }$), i.e. events with two photons in the annihilation window $[460\, \text{ keV }, 545\, \text{ keV}]$ and one photon in the prompt energy window of $[568\, \text{ keV },639\, \text{ keV}]$ (see Fig. 2 of Ref. [25] for the energy spectrum for ^124^I measured on Quadra). ^124^I has the convenient property of having a prompt photon with an energy of $602.73 \pm 0.08 \, \text{ keV }$, which Quadra’s detector can fully resolve. The seemingly high prompt photon branching ratio (BR) of $62.9 \pm 0.7 \,\%$ [39, 40] reduces to $12.0 \pm 1.1\,\%$ per positron, i.e. when electron capture decays are removed. The spatial location of a $3\gamma \text{ E }$ is determined through the time-of-flight (TOF) information of the two annihilation photons, i.e. there is no image reconstruction along the line of Refs. [41–45]. The histoimages of $3\gamma \text{ E }$ are binned into three different voxel sizes of $10.0\times 10.0 \times 10.0 \, \text{ mm}^3$, $7.1\times 7.1 \times 7.1 \, \text{ mm}^3$ and $4.0\times 4.0 \times 4.0 \, \text{ mm}^3$, respectively. We consider the $10\times 10\times 10 \, \text{ mm}^3$ voxel size to be on the verge of clinical usefulness. On the other end, we chose the smallest voxel size such that it would really push oPs imaging on Quadra to its limits. According to Refs. [46, 47], $4\,\text{ mm }$ is about the spatial resolution that is achievable in coincidence PET imaging with ^124^I. Finally, $7.1\times 7.1\times 7.1 \, \text{ mm}^3$ sits in the middle and could be thought of as similar to the spatial resolution of a SPECT/CT system. As a comparison, we also perform a fit that encompasses all $3\gamma \text{ E }$ in a single tube.

The time difference distributions (TDD) are the binned time differences between the annihilation and prompt photons in each voxel (time bin width is $133\, \text{ ps }$, i.e. slightly above Quadras time resolution). We use the Bayesian fitting procedure discussed in Refs. [24, 25] to determine the oPs lifetime $\tau _{3}$ from a measured TDD. The fitting model is the same as in Refs. [24, 25], i.e. a Gaussian function convoluted with three lifetime components for pPs, direct annihilation and oPs. In contrast to Ref. [24], we fix the pPs lifetime $\tau _1 = 125 \,\text{ ps }$ and direct annihilation $\tau _2= 388\, \text{ ps }$ together with the background count number. The background is fixed as the mean value of time differences that are smaller than $-2.5 \, \text{ ns }$. We fit the following priors with a Gaussian likelihood to the voxel-wise TDD

1 $$\begin{aligned}&\tau _{3} \sim \mathcal {N}(1.78 \, \text{ ns }, 0.8\, \text{ ns}) \;, \\&BR_{1,2,3} \; \sim \; \textrm{Dirichlet}(0.75, 3.1, 1.15) \;,\\&\sigma \sim \mathcal {N}(0.1 \,\text{ ns }, 0.05 \,\text{ ns}) \,, \\&\Delta \sim \mathcal {N}(0 \,\text{ ns }, 0.5 \,\text{ ns}) \;, \\&N \; \sim \; \mathcal {N}(A, 0.1\cdot A) \quad \text{ with } \quad A \; = \;\int dt \, \left( y_i - b \right) \;, \end{aligned}$$

where *b* is the background value and $y_i$ are the bin values of the TDD. In the oPs lifetime images, we only selected voxels that have a relative error in the background region of less than $20\%$. We fit time differences in the range from $-2\,\text{ ns }$ to $8\,\text{ ns }$.

The posterior distribution of $\tau _{3}$ is bell-shaped with hardly any skewness. We therefore report the uncertainty of $\tau _{3}$ with a standard deviation, which is estimated with the common point estimate. The uncertainty of the BR $BR_{1,2,3}$ is given in terms of the $68\%$ highest density intervals (HDI) of the posterior distribution since a point estimate of the standard deviation would not make sense for a Dirichlet variable.

## Results

**Fig. 4:**
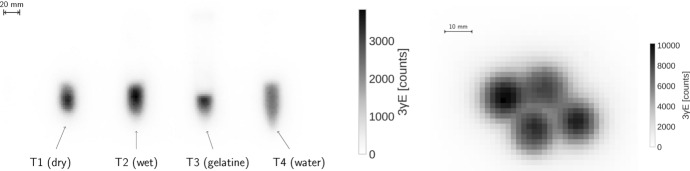
Side-view MIP of the histoimage of the separated tubes (left) and top-viw MIP of the tubes taped together (right)

The two panels in Fig. 4 show the MIP of the histoimage, i.e. the number of identified $3\gamma \text{ E }$. from the side (left panel) and from the top (right panel) for the two experimental setups. For both histoimages the voxel size is $1.9 \times 1.9 \times 1.65 \, \textrm{mm}^3$.

**Fig. 5:**
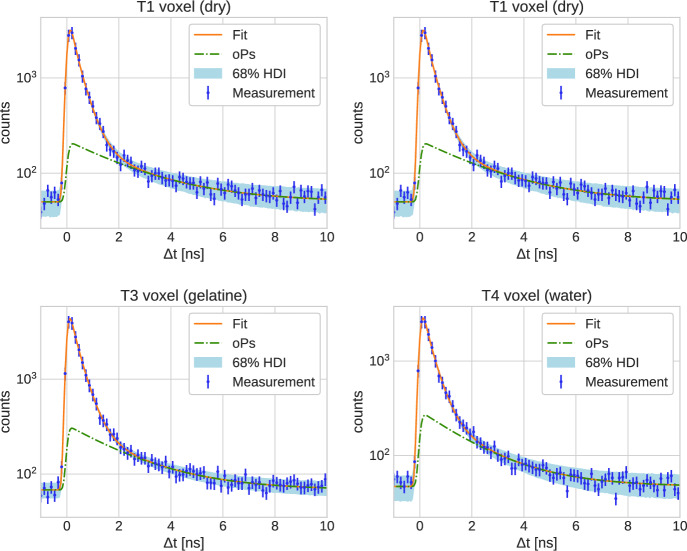
Single-voxel TDD together with the fit prediction, posterior 68% HDI and oPs component in logarithmic scale ($4\times 4\times 4 \, \text{ mm}^3$ voxel size). The fit results are reported in Table 2

The top four rows of Table 2 report the single tube fits, i.e. when all measured time differences in a tube are collected in one TDD (no spatial binning of the $3\gamma \text{ E }$ data). Clearly, the different humidity levels of the XAD4 powder lead to significantly distinct oPs lifetimes. Furthermore, Table 2 includes also the results from fitting a TDD from a single $4\times 4\times 4 \, \text{ mm}^3$ voxel. The voxel is chosen in the central region of each tube. This allows us to get a good intuition about the count statistics for the smallest voxel size. Figure 5 shows the corresponding single-voxel TDD together with the fit prediction for the oPs lifetime component.

**Table 2:** Fit results for the full samples (top four rows) and single voxel with a size of $4\times 4\times 4 \, \text{ mm}^3$ (bottom four rows)

Sample	$\tau _{3} \, [\text{ ns}]$	$BR_1$	$\text{ HDI}_{BR_1}$	$BR_2$	$\text{ HDI}_{BR_2}$	$BR_3$	$\text{ HDI}_{BR_3}$
T1 (dry)	2.52 ± 0.03	0.073	[0.071, 0.074]	0.716	[0.714, 0.718]	0.211	[0.21, 0.213]
T2 (wet)	2.37 ± 0.03	0.081	[0.079, 0.082]	0.702	[0.7, 0.704]	0.217	[0.216, 0.219]
T3 (gelatine)	2.27 ± 0.04	0.073	[0.071, 0.075]	0.705	[0.702, 0.708]	0.222	[0.22, 0.223]
T4 (water)	1.82 ± 0.02	0.088	[0.087, 0.09]	0.644	[0.641, 0.646]	0.268	[0.267, 0.269]
T1 voxel	2.56 ± 0.23	0.09	[0.081, 0.099]	0.702	[0.687, 0.716]	0.208	[0.2, 0.216]
T2 voxel	2.37 ± 0.23	0.073	[0.062, 0.085]	0.69	[0.671, 0.708]	0.237	[0.227, 0.247]
T3 voxel	2.3 ± 0.12	0.044	[0.039, 0.05]	0.745	[0.736, 0.754]	0.21	[0.205, 0.215]
T4 voxel	2.0 ± 0.14	0.062	[0.052, 0.072]	0.683	[0.666, 0.7]	0.255	[0.246, 0.264]

**Fig. 6:**
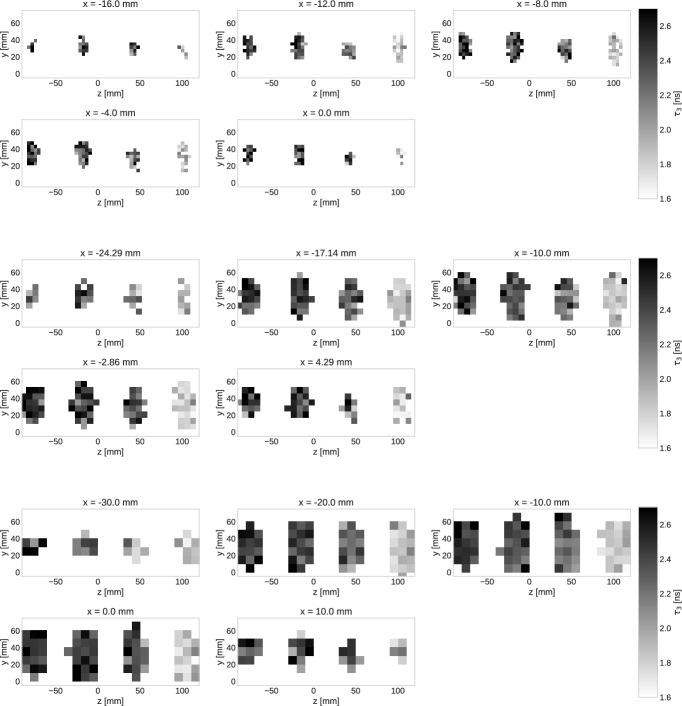
Slices of the oPs lifetime images along the *x*-axis for the separated tubes. The voxel size is increasing from top to bottom

**Fig. 7:**
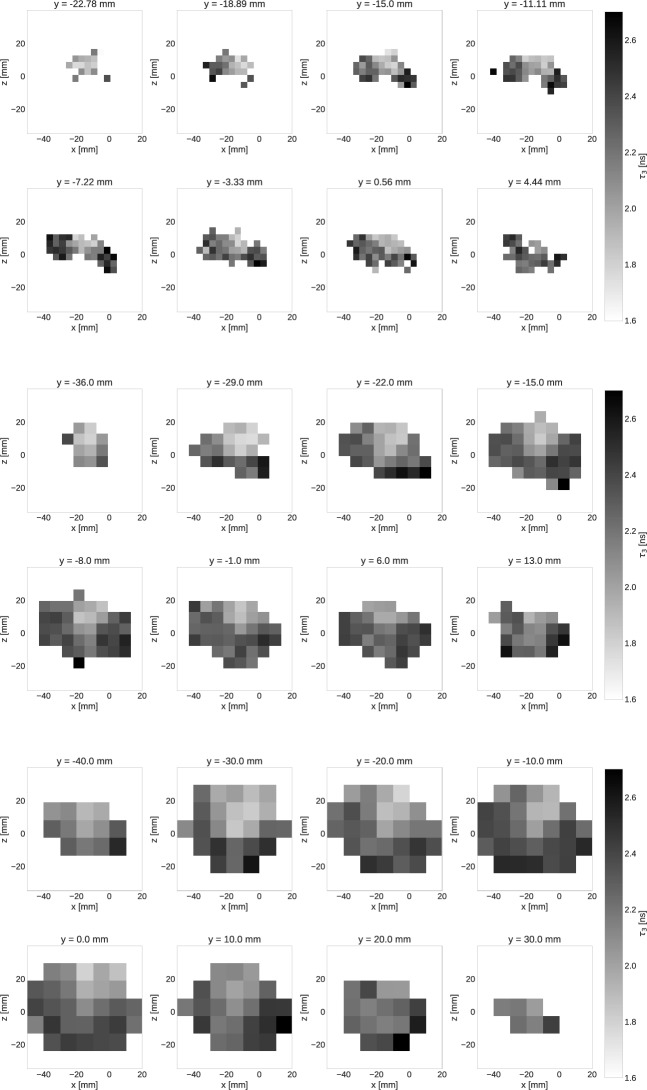
Slices of the oPs lifetime images along the *y*-axis for the tubes taped together. The voxel size is increasing from top to bottom

In Figs. 6 and 7 we show 2D slices of the oPs lifetime images for the two scans with separated and taped-together tubes. The two top rows show slices for the $4\times 4\times 4 \, \text{ mm}^3$ voxel size, while the middle and bottom rows are for the $7.1\times 7.1\times 7.1 \, \text{ mm}^3$ and $10\times 10\times 10 \, \text{ mm}^3$ voxel sizes, respectively. For best visualization, the slices of the separated tubes in Fig. 6 are shown in the $z-y$ plane, analogously to a coronal PET MIP in Fig. 2. For the tubes close together, we chose the $x-y$ plane as in the CT slice in Fig. 3. No post-processing, such as smoothing or any filtering, was applied to the oPs lifetime images.

**Fig. 8:**
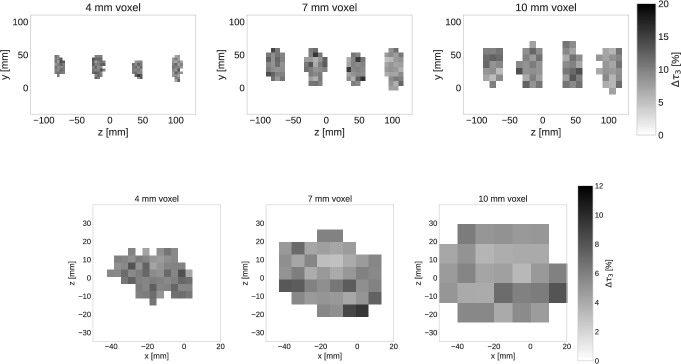
MIP of the relative uncertainties of $\tau _{3}$ for the two separated (top) and taped together (bottom) experimental setups

Finally, we show the MIP of the standard deviation for $\tau _{3}$ for the three voxel sizes and the two experimental setups in Fig. 8. The color bar in these figures shows the relative uncertainty of $\tau _{3}$ for each voxel.

## Discussion

First, we would like to highlight the low statistical uncertainty of the oPs lifetime in the single tube fit in Table 2. Within a few ml, an activity concentration low as $232 \, \text{ kBq }/\text{ml }$ and a scan time of 15 min, the marginalized posterior distribution of $\tau _{3}$ has a relative standard deviation of less than $1.76\%$ using a commercial LAFOV PET/CT and Ref. [25]’s methodology. Apart from the higher prompt photon BR per positron of ^124^I compared to ^68^Ga  the main advantage of ^124^I is Quadra’s capability to resolve ^124^I’s photopeak and thereby increasing the peak signal-to-background ratio (pSBR). In addition, the smaller decay constant ^124^I allows for a longer scan time and larger time-integrated counts compared to ^82^Rb (which has a comparable prompt photon BR per positron with ^124^I). This high statistical precision allows to distinguish the oPs lifetime in the four samples, confirming Ref. [36] in that XAD4 is well suited for performance evaluations and intercomparisons of PET/CT scanners for oPs lifetime imaging.

In the literature, oPs lifetime imaging has shown to be feasible only with ^22^Na and very long scan times and/or large voxel sizes. E.g. Ref. [15] applied a spatial binning of $2\times 2\times 2 \, \text{ cm }$ to their tissue sample data. Likewise, Ref. [18] seems to have a voxel size of multiple cm. It remains unclear, how much viable clinical information such voxel sizes may contain. With ^82^Rb and ^68^Ga, i.e. radionuclides that are used in clinical routine, the count statistics and in particular the pSBR are not sufficient for oPs lifetime imaging, given our methodology. This is why in Refs. [24, 25] we refrained from performing voxel-wise oPs lifetime fits.

Considering Fig. 6, it is clear that even with the $4\times 4\times 4 \, \text{ mm}^3$ the oPs lifetime in the four tubes are distinguishable. From left to right, $\tau _{3}$ decreases according to the sample filling. Homogeneity of $\tau _{3}$, i.e. of the gray scale, within a single tube clearly gets worse with decreasing voxel size, which simply reflects the decreasing count statistics within a voxel (see also Fig. 8). The error of $\tau _{3}$ increases towards the tube walls, but as Fig. 8 shows, the relative error still remains around $10\%$. Relaxing the $20\%$ background error condition for fitting voxels could possibly provide a shape of the oPs lifetime images that is more consistent with the real shape of the tubes.

With the second experimental setup, i.e. with the tubes tied together, we wanted to create a very challenging situation for oPs lifetime imaging. Indeed, the top view slices in Fig. 7 do not allow for a clear spatial distinction of the four tubes. With respect to the oPs lifetimes, the shorter $\tau _{3}$ in the water tube can be best distinguished from the other tubes in the $7.1\times 7.1\, \text{ mm}^2$ slices. Also in the smallest voxel size, the light gray area of T4 is distinguishable. However, without knowing anything about the experimental setup, i.e. without having seen Figs. 2 and 3, it would be impossible to interpret Fig. 7 as the four tubes with different oPs lifetimes. An improved visualization could easily be achieved through a post-processing of the images, like e.g. interpolating the voxel values, resampling to smaller voxel values or by an overlay with the CT image. We deliberately refrain from any form of image post-processing for Fig. 7 in order to explicitly show the limitation of oPs lifetime imaging with Quadra. The longer scan of this setup compared to the separated tubes (40 min vs. 15 min) is noticeable in the lower $\tau _{3}$ error, as shown in Fig. 8. However, the limiting factor for this experiment is the localization of the $3\gamma \text{ E }$  which relies solely on TOF. The right panel of Fig. 4 shows that although the tubes are distinguishable, there is a significant blurring of the tubes.

An enhancement of Fig. 7 without post-processing would probably require a smaller voxel size (as in Fig. 4 but at a comparable level of count statistics. The recently proposed reconstruction methods from Refs. [28, 41, 44, 45] suggest that voxel sizes smaller than $4\,\text{ mm }$ should be feasible. Certainly, the improved $3\gamma \text{ E }$ selection and localization of Refs. [41, 44] would allow for a lower background of random events, i.e. higher pSBR. The application of these algorithms to Quadra data is left for future studies.

In view of clinical applications, a certain level post-processing of the oPs lifetime images would improve the diagnostic value of such an image, given the current methodology. Simplifying the physical model for fitting the oPs lifetime, as done e.g. in Refs. [18, 28] can boost the count statistics, however it would be difficult to compare with reference values in the literature (and the interpretation in terms of oPs lifetime might be hampered). Future studies will certainly show how much a more sophisticated event selection and reconstruction algorithm can improve on the results presented here.

It should be mentioned, that the activity concentration in the four samples (see Table 1) is still somewhat higher than one could expect in a thyroid cancer patient (though $\textrm{SUVmax}\gtrsim 1000$ have been reported for lesions in thyroid cancer patients). In our study, we did not investigate possible effects that may increase the number of random events when performing human scans. These might include attenuation, scatter, high activity in the FOV, scatter effects etc.

## Conclusions

This brief report demonstrates that oPs lifetime imaging, achieved as a 3D image with $\tau _{3}$ as voxel values, is feasible using a commercial PET/CT scanner under conditions that would be viable for a thyroid patient scan with respect to the isotope, activity concentration, scan time and voxel sizes. The Quadra scanner, in combination with our data analysis methodology, is able to capture oPs lifetimes with notable precision, even at voxel sizes as small as $4.0^3\, \text{ mm}^3$. These results affirm that the Quadra can yield distinct, voxel-wise lifetime measurements across various sample compositions, enabling diagnostic-level imaging using ^124^I-based compounds. Future work could focus on advanced reconstruction algorithms and smoothing techniques, potentially enhancing both the diagnostic utility and spatial resolution of oPs lifetime images, especially in challenging setups with closely positioned samples.

## Data Availability

Evaluated data are available in the Zenodo repository https://doi.org/10.5281/zenodo.13443797.

## References

[1] Bass SD, Mariazzi S, Moskal P, Stępień EŁ. Colloquium: positronium physics and biomedical applications. Rev Mod Phys. 2023;95:021002. 10.1103/RevModPhys.95.021002.

[2] Vértes A, Nagy S, Klencsár Z, Lovas RG, Rösch F. Handbook of Nuclear Chemistry: Vol. 1: Basics of Nuclear Science. Handbook of Nuclear Chemistry. Springer New York, USA, 2010. 10.1007/978-1-4419-0720-2

[3] Moskal P, Jasińska B, Stępień EŁ, Bass SD. Positronium in medicine and biology. Nat Rev Phys. 2019;1(9):527–9. 10.1038/s42254-019-0078-7.

[4] Hourlier A, Boisson F, Brasse D. Experimental uses of positronium and potential for biological applications. IEEE Trans Radiat Plasma Med Sci. 2024;8(6):581–94. 10.1109/TRPMS.2024.3407981.

[5] Shibuya K, Saito H, Nishikido F, Takahashi M, Yamaya T. Oxygen sensing ability of positronium atom for tumor hypoxia imaging. Commun Phys. 2020;3:173. 10.1038/s42005-020-00440-z.

[6] Moskal P, Stępien EŁ. Positronium as a biomarker of hypoxia. Bio-Algorithms Med-Syst. 2021;17(4):311–9. 10.1515/bams-2021-0189.

[7] Moskal P, Stępień EŁ. Prospects and clinical perspectives of total-body PET imaging using plastic scintillators. PET Clinics. 2020;15(4):439–52. 10.1016/j.cpet.2020.06.009. 32739047 10.1016/j.cpet.2020.06.009

[8] Moskal P, Stępień EŁ. Perspectives on translation of positronium imaging into clinics. Front Phys. 2022;10:969806. 10.3389/fphy.2022.969806.

[9] Moskal P, Kisielewska D, Curceanu C, Czerwiński E, Dulski K, Gajos A, Gorgol M, Hiesmayr B, Jasińska B, Kacprzak K, Kapłon Ł, Korcyl G, Kowalski P, Krzemień W, Kozik T, Kubicz E, Mohammed M, Niedźwiecki S, Pałka M, Pawlik-Niedźwiecka M, Raczyński L, Raj J, Sharma S, Shivani SRY, Silarski M, Skurzok M, Stępień E, Wiślicki W, Zgardzińska B. Feasibility study of the positronium imaging with the J-PET tomograph. Phys Med Biol. 2019;64:055017. 10.1088/1361-6560/aafe20. 30641509 10.1088/1361-6560/aafe20

[10] Tashima H, Yamaya T. Three-gamma imaging in nuclear medicine: a review. IEEE Trans Radiat Plasma Med Sci. 2024;8(8):853–66. 10.1109/trpms.2024.3470836.

[11] Stepanov PS, Selim FA, Stepanov SV, Bokov AV, Ilyukhina OV, Duplâtre G, Byakov VM. Interaction of positronium with dissolved oxygen in liquids. Phys Chem Chem Phys. 2020;22:5123–31. 10.1039/c9cp06105c. 32073009 10.1039/c9cp06105c

[12] Stepanov SV, Byakov VM, Stepanov PS. Positronium in biosystems and medicine: a new approach to tumor diagnostics based on correlation between oxygenation of tissues and lifetime of the positronium atom. Phys Wave Phenomena. 2021;29:174–9. 10.3103/S1541308X21020138.

[13] Takyu S, Matsumoto K-I, Hirade T, Nishikido F, Akamatsu G, Tashima H, Takahashi M, Yamaya T. Quantification of radicals in aqueous solution by positronium lifetime: an experiment using a clinical PET scanner. Jpn J Appl Phys. 2024;63(8):086003. 10.35848/1347-4065/ad679a.

[14] Takyu S, Nishikido F, Tashima H, Akamatsu G, Matsumoto K-I, Takahashi M, Yamaya T. Positronium lifetime measurement using a clinical pet system for tumor hypoxia identification. Nucl Instrum Methods Phys Res, Sect A. 2024;1065:169514. 10.1016/j.nima.2024.169514.

[15] Moskal P, Dulski K, Chug N, Curceanu C, Czerwiński E, Dadgar M, Gajewski J, Gajos A, Grudzień G, Hiesmayr BC, Kacprzak K, Kapłon Ł, Karimi H, Klimaszewski K, Korcyl G, Kowalski P, Kozik T, Krawczyk N, Krzemień W, Kubicz E, Małczak P, Niedźwiecki S, Pawlik-Niedźwiecka M, Pędziwiatr M, Raczyński L, Raj J, Ruciński A, Sharma S, Shivani Shopa RY, Silarski M, Skurzok M, Stępień EŁ, Szczepanek M, Tayefi F, Wiślicki W. Positronium imaging with the novel multiphoton PET scanner. Sci Adv. 2019;7:4394. 10.1126/sciadv.abh4394. 34644101 10.1126/sciadv.abh4394 PMC11559468

[16] Karimi H, Moskal P, Żak A, Stępień EŁ. 3D melanoma spheroid model for the development of positronium biomarkers. Sci Rep. 2023;13(1):7648. 10.1038/s41598-023-34571-4. 37169794 10.1038/s41598-023-34571-4 PMC10175546

[17] Moskal P, Kubicz E, Grudzień G, Czerwiński E, Dulski K, Leszczyński B, Niedźwiecki S, Stȩpień EŁ. Developing a novel positronium biomarker for cardiac myxoma imaging. EJNMMI Phys. 2023;10:22. 10.1186/s40658-023-00543-w. 36959477 10.1186/s40658-023-00543-w PMC10036702

[18] Moskal P, Baran J, Bass S, Choiński J, Chug N, Curceanu C, Czerwiński E, Dadgar M, Das M, Dulski K, Eliyan KV, Fronczewska K, Gajos A, Kacprzak K, Kajetanowicz M, Kaplanoglu T, Kapłon Ł, Klimaszewski K, Kobylecka M, Korcyl G, Kozik T, Krzemień W, Kubat K, Kumar D, Kunikowska J, Mşczewska J, Migdał W, Moskal G, Mryka W, Niedźwiecki S, Parzych S, Rio EP, Raczyński L, Sharma S, Shivani S, Shopa RY, Silarski M, Skurzok M, Tayefi F, Ardebili KT, Tanty P, Wiślicki W, Królicki L, Stępień EŁ. Positronium image of the human brain in vivo. Sci Adv. 2024;10(37):2840. 10.1126/sciadv.adp2840. 39270027 10.1126/sciadv.adp2840 PMC11397496

[19] Avachat AV, Mahmoud KH, Leja AG, Xu JJ, Anastasio MA, Sivaguru M, Di Fulvio A. Ortho-positronium lifetime for soft-tissue classification. Sci Rep. 2024;14(1):21155. 10.1038/s41598-024-71695-7. 39256482 10.1038/s41598-024-71695-7 PMC11387643

[20] Shimazoe K, Uenomachi M. Multi-molecule imaging and inter-molecular imaging in nuclear medicine. Bio-Algorithms Med-Syst. 2022;18(1):127–34. 10.2478/bioal-2022-0081.

[21] Shimazoe K, Uenomachi M, Takahashi H. Imaging and sensing of pH and chemical state with nuclear-spin-correlated cascade gamma rays via radioactive tracer. Commun Phys. 2022;5(1):24. 10.1038/s42005-022-00801-w.

[22] Zaleski R, Kotowicz O, Górska A, Zaleski K, Zgardzińska B. Investigation of the ability to detect electrolyte disorder using PET with positron annihilation lifetime spectroscopy. J Phys Chem B. 2023;127(46):9887–90. 10.1021/acs.jpcb.3c04208. 37946359 10.1021/acs.jpcb.3c04208 PMC10683008

[23] Shimazoe K, Donghwan K, Mineo T, Sato T, Ohta S, Tatsumi T, Sugiyama A, Yamatsugu K, Nomura S, Terabayashi R. pH dependence of perturbed angular correlation in DOTA chelated 111 In measured with ring-shape gamma-ray detectors. Interactions. 2024;245(1):22. 10.1007/s10751-024-01864-7.

[24] Mercolli L, Steinberger WM, Sari H, Afshar-Oromieh A, Caobelli F, Conti M, Felgosa Cardoso ÂR, Mingels C, Moskal P, Pyka T, Rathod N, Schepers R, Seifert R, Shi K, Stępień EŁ, Viscione M, Rominger AO. In vivo positronium lifetime measurements with a long axial field-of-view pet/ct. medRxiv 2024. 10.1101/2024.10.19.24315509

[25] Steinberger WM, Mercolli L, Breuer J, Sari H, Parzych S, Niedzwiecki S, Lapkiewicz G, Moskal P, Stępień EŁ, Rominger A, Shi K, Conti M. Positronium lifetime validation measurements using a long-axial field-of-view positron emission tomography scanner. EJNMMI Phys. 2024;11:76. 10.1186/s40658-024-00678-4. 39210079 10.1186/s40658-024-00678-4 PMC11362402

[26] Prenosil GA, Sari H, Fürstner M, Afshar-Oromieh A, Shi K, Rominger A, Hentschel M. Performance characteristics of the Biograph Vision Quadra PET/CT system with a long axial field of view using the NEMA NU 2–2018 standard. J Nucl Med. 2022;63:476–84. 10.2967/jnumed.121.261972. 34301780 10.2967/jnumed.121.261972

[27] Spencer BA, Berg E, Schmall JP, Omidvari N, Leung EK, Abdelhafez YG, Tang S, Deng Z, Dong Y, Lv Y, Bao J, Liu W, Li H, Jones T, Badawi RD, Cherry SR. Performance evaluation of the uEXPLORER total-body PET/CT scanner based on NEMA NU 2–2018 with additional tests to characterize PET scanners with a long axial field of view. J Nucl Med. 2021;62:861–70. 10.2967/jnumed.120.250597. 33008932 10.2967/jnumed.120.250597 PMC8729871

[28] Chen Z, Kao C-M, Huang H-H, An L. Enhanced positronium lifetime imaging through two-component reconstruction in time-of-flight positron emission tomography. Front Phys. 2024;12:1429344. 10.3389/fphy.2024.1429344. 10.3389/fphy.2024.1429344 PMC12829466 41585607

[29] Moskal P, Kisielewska D, Shopa RY, Bura Z, Chhokar J, Curceanu C, Czerwiński E, Dadgar M, Dulski K, Gajewski J, Gajos A, Gorgol M, Del Grande R, Hiesmayr BC, Jasińska B, Kacprzak K, Kamińska A, Kapłon Ł, Karimi H, Korcyl G, Kowalski P, Krawczyk N, Krzemień W, Kozik T, Kubicz E, Małczak P, Mohammed M, Niedźwiecki S, Pałka M, Pawlik-Niedźwiecka M, Pędziwiatr M, Raczyński L, Raj J, Ruciński A, Sharma S, Shivani S, Silarski M, Skurzok M, Stępień E.Ł, Vandenberghe S, Wiślicki W, Zgardzińska B. Performance assessment of the 2 gamma positronium imaging with the total-body PET scanners. EJNMMI Phys 2020;7(1):44 10.1186/s40658-020-00307-w 10.1186/s40658-020-00307-w PMC7326848 32607664

[30] Moskal P. Positronium Imaging. In: 2019 IEEE Nuclear Science Symposium and Medical Imaging Conference (NSS/MIC), 2019; pp 1–3. 10.1109/NSS/MIC42101.2019.9059856

[31] Shopa RY, Dulski K. Positronium imaging in J-PET with an iterative activity reconstruction and a multi-stage fitting algorithm. Bio-Algorithms Med-Syst. 2023;19(1):54–63.

[32] Takyu S, Ikeda H, Wakizaka H, Nishikido F, Matsumoto K-I, Tashima H, Suzuki H, Funaki Y, Watabe H, Takahashi M, Yamaya T. Positron annihilation lifetime measurement with TOF-PET detectors: feasibility of Iodine-124 use. Appl Phys Express. 2023;16(11):116001. 10.35848/1882-0786/ad047c.

[33] Plyku D, Hobbs RF, Wu D, Garcia C, Sgouros G, Van Nostrand D. I-124 PET/CT image-based dosimetry in patients with differentiated thyroid cancer treated with I-131: correlation of patient-specific lesional dosimetry to treatment response. Ann Nucl Med. 2022;36(3):213–23. 10.1007/s12149-021-01655-y. 35119623 10.1007/s12149-021-01655-y

[34] Dittmann M, Gonzalez Carvalho JM, Rahbar K, Schäfers M, Claesener M, Riemann B, Seifert R. Incremental diagnostic value of [18F]tetrafluoroborate PET-CT compared to [131I]iodine scintigraphy in recurrent differentiated thyroid cancer. Eur J Nucl Med Mol Imaging. 2020;47(11):2639–46. 10.1007/s00259-020-04727-9. 32248325 10.1007/s00259-020-04727-9 PMC7515952

[35] Ventura D, Dittmann M, Büther F, Schäfers MA. Diagnostic performance of [18F]TFB PET/CT compared with therapeutic activity [131I]Iodine SPECT/CT and [18F]FDG PET/CT in recurrent differentiated thyroid carcinoma. J Nucl Med. 2023;65(2):192–8. 10.2967/jnumed.123.266513. 10.2967/jnumed.123.266513 PMC10858375 38164565

[36] Łapkiewicz G, Niedźwiecki S, Moskal P. Developing a phantom for the positronium imaging evaluation. Acta Phys Polonica B Proc Supple. 2022;15(4):10. 10.5506/APhysPolBSupp.15.4-A4.

[37] Kotera K, Saito T, Yamanaka T. Measurement of positron lifetime to probe the mixed molecular states of liquid water. Phys Lett A. 2005;345(1):184–90. 10.1016/j.physleta.2005.07.018.

[38] Jentzen W, Freudenberg L, Eising EG, Sonnenschein W, Knust J, Bockisch A. Optimized 124I PET dosimetry protocol for radioiodine therapy of differentiated thyroid cancer. J Nucl Med. 2008;49(6):1017–23. 10.2967/jnumed.107.047159. 18483099 10.2967/jnumed.107.047159

[39] Katakura J-I, Wu ZD. Nuclear data sheets for A=124. Nucl Data Sheets. 2008;109(7):1655–877. 10.1016/j.nds.2008.06.001.

[40] Das M, Mryka W, Beyene EY, Parzych S, Sharma S, Stępień EŁ, Moskal P. Estimating the efficiency and purityfor detecting annihilation and promptphotons for positronium imagingwith j-pet using toy monte carlosimulation. Bio-Algorithms Med-Syst. 2023;19(1):87–95. 10.5604/01.3001.0054.1938.

[41] Qi J, Huang B. Positronium lifetime image reconstruction for TOF PET. IEEE Trans Med Imaging. 2022;41:2848–55. 10.1109/TMI.2022.3174561. 35584079 10.1109/TMI.2022.3174561 PMC9829407

[42] Shibuya K, Saito H, Tashima H, Yamaya T. Using inverse Laplace transform in positronium lifetime imaging. Phys Med Biol. 2022;67(2):025009. 10.1088/1361-6560/ac499b. 10.1088/1361-6560/ac499b 35008076

[43] Chen Z, An L, Kao C-M, Huang H-H. The properties of the positronium lifetime image reconstruction based on maximum likelihood estimation. Bio-Algorithms Med-Syst. 2023;19(1):1–8. 10.5604/01.3001.0054.1807. 10.5604/01.3001.0054.1807 PMC12829913 41586385

[44] Huang B, Li T, Arino-Estrada G, Dulski K, Shopa RY, Moskal P, Stepien E, Qi J. SPLIT: statistical positronium lifetime image reconstruction via time-thresholding. IEEE Trans Med Imaging. 2024;43:2148–58. 10.1109/TMI.2024.3357659. 38261489 10.1109/TMI.2024.3357659 PMC11409919

[45] Jegal J, Jeong D, Seo E-S, Park H, Kim H. Convolutional neural network-based reconstruction for positronium annihilation localization. Sci Rep. 2022;12:8531. 10.1038/s41598-022-11972-5. 35595738 10.1038/s41598-022-11972-5 PMC9122910

[46] Kertész H, Conti M, Panin V, Cabello J, Bharkhada D, Beyer T, Papp L, Jentzen W, Cal-Gonzalez J, Herraiz JL, López-Montes A, Rausch I. Positron range in combination with point-spread-function correction: an evaluation of different implementations for [124I]-PET imaging. EJNMMI Phys. 2022;9(1):56. 10.1186/s40658-022-00482-y. 35984531 10.1186/s40658-022-00482-y PMC9391565

[47] Kersting D, Moraitis A, Sraieb M, Zarrad F, Umutlu L, Rischpler C, Fendler WP, Herrmann K, Weber M, Conti M, Fragoso Costa P, Jentzen W. Quantification performance of silicon photomultiplier-based PET for small 18F-, 68Ga- and 124I-avid lesions in the context of radionuclide therapy planning. Physica Med. 2023;114:103149. 10.1016/j.ejmp.2023.103149. 10.1016/j.ejmp.2023.103149 37778973

