# Correction: Phantom imaging demonstration of positronium lifetime with a long axial field‑of‑view PET/CT and ^124^I

**DOI:** 10.1186/s40658-025-00801-z

**Published:** 2025-09-17

**Authors:** Lorenzo Mercolli, William M. Steinberger, Narendra Rathod, Maurizio Conti, Paweł Moskal, Axel Rominger, Robert Seifert, Kuangyu Shi, Ewa Ł. Stępień, Hasan Sari

**Affiliations:** 1 Department of Nuclear Medicine, Inselspital, Bern University Hostpital, University of Bern, Bern, Switzerland; 2 ARTORG Center for Biomedical Engineering Research, University of Bern, Bern, Switzerland; 3 Albert Einstein Center for Fundamental Physics (AEC), Laboratory for High Energy Physics (LHEP), University of Bern, Bern, Switzerland; 4 Siemens Medical Solutions USA, Inc, Knoxville, TN USA; 5 Faculty of Physics, Astronomy and Applied Computer Science, Jagiellonian University, Krakow, Poland; 6 Centre for Theranostics, Jagiellonian University, Krakow, Poland; 7 Siemens Healthineers International AG, Zürich, Switzerland

**Correction: EJNMMI Phys 12, 80 (2025)**

10.1186/s40658-025-00790-z

Following publication of the original article [1], Table 1 and its caption has been updated from:

**Table 1:**
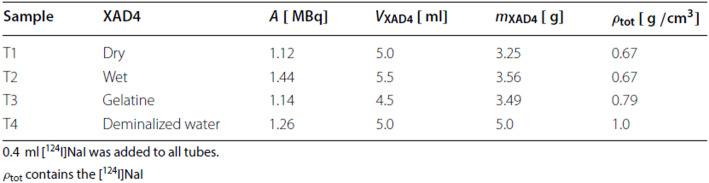
Summary of the sample preparation

To:

**Table 1:**
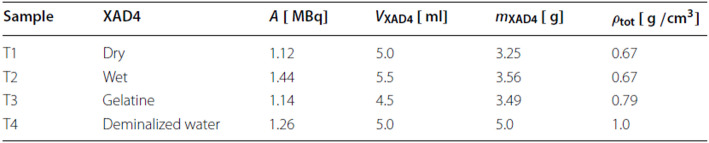
Summary of the sample preparation. 0.4 Ml [^124^I]NaI was added to all tubes. *ρ*tot contains the [^124^I]NaI

The original article [1] has been updated.
